# Intergenerational Transmission of Parental Favoritism and Disfavoritism in Middle and Later Adulthood

**DOI:** 10.1093/geroni/igaf122.1595

**Published:** 2025-12-31

**Authors:** J Jill Suitor, Megan Gilligan, Robert Frase, Destiny Ogle, Ranran He, Yifei Hou

**Affiliations:** Purdue University, West Lafayette, Indiana, United States; University of Missouri, Columbia, Missouri, United States; Southern Illinois University, Carbondale, Illinois, United States; Purdue University, West Lafayette, Indiana, United States; Purdue University, West Lafayette, Indiana, United States; School of International and Public Affairs, Shanghai, Shanghai, China (People’s Republic)

## Abstract

Drawing from theories of socialization and agency, we use mixed-method data on 187 multigenerational families from the Within-Family Differences Study to examine whether midlife sons’ and daughters’ (G2) self-perceptions of their mothers’ (G1) favoritism and disfavoritism shape their own patterns of differentiation among their offspring (G3). Prior research has suggested that parents’ differential provision of support may be transmitted intergenerationally. The present study extends this work by focusing on the role of adult children’s self-perceptions of their mothers’ relational favoritism and disfavoritism in shaping their likelihood of differentiating among their own offspring. We study these processes late in the life course when G1 mothers are in their late 80s and 90s, and their children are in later midlife—a point by which it might be expected that differentiation among offspring would be less common. We concentrate on dimensions of relational differentiation that we have found to have the greatest impact on adult children’s well-being—emotional closeness, conflict, and disappointment. Our findings revealed that adult children (G2) who perceived that their mothers (G1) currently differentiated on these relational dimensions were more likely to differentiate among their own offspring (G3) on the same dimensions. Further, patterns of differentiation in both generations appeared to be fueled by the same structural and socioemotional factors, with children’s gender, perceived value similarity, and children’s violations of expectations shaping which offspring were favored and disfavored. Thus, the findings indicate that both patterns and predictors of relational favoritism and disfavoritism are similar across generations.

